# Migrant workers occupational health research: an OMEGA-NET working group position paper

**DOI:** 10.1007/s00420-021-01803-x

**Published:** 2021-10-18

**Authors:** Emine Aktas, Barbara Bergbom, Lode Godderis, Bertina Kreshpaj, Mario Marinov, Dana Mates, Damien M. McElvenny, Ingrid Sivesind Mehlum, Valentina Milenkova, Evangelia Nena, Deborah C. Glass

**Affiliations:** 1 Florence Nightingale Faculty of Nursing, Department of Public Health Nursing, Istanbul University-Cerrahpasa, Istanbul, Turkey; 2 Department of Public Health and Primary Care, Centre for Environment & Health, KU Leuven, Leuven, Belgium; 3 Finnish Institute of Occupational Health, Helsinki, Finland; 4 External Service for Prevention and Protection at Work, IDEWE, Heverlee, Belgium; 5 Unit of Occupational Medicine, Institute of Environmental Medicine, Karolinska Institutet, Solna, Sweden; 6 South-West University “Neofit Rilski”, Blagoevgrad, Bulgaria; 7 The National Institute of Public Health, Bucharest, Romania; 8 Institute of Occupational Medicine, Edinburgh, UK; 9 University of Manchester, Manchester, UK; 10 National Institute of Occupational Health, Oslo, Norway; 11 Institute of Health and Society, University of Oslo, Oslo, Norway; 12 Medical School, Democritus University of Thrace, Alexandroupolis, Greece; 13 MonCOEH, Monash University, 553 ST Kilda Road, Melbourne, 3004 Australia

**Keywords:** Migrant workers, Native workers, Occupational health, Study design, Working conditions, Work-related health

## Abstract

**Objective:**

The aims of the study were: (1) to clarify the definitions of “migrant” used in occupational health research; (2) to summarize migrant workers’ industry sectors, occupations and employment conditions; (3) to identify the occupational health and safety services available to migrant workers; (4) to summarize work-related health problems found among migrant workers; (5) to identify the methodological challenges to research into occupational health of migrant workers; and (6) to recommend improvements in migrant occupational health research.

**Methods:**

This position paper was prepared by researchers from several European countries and Australia, working within the EU COST Action OMEGA-NET. The paper drew on two recent systematic reviews on the occupational health of international migrant workers and other literature, and also identified uncertainties and gaps in the research literature. Migrants may, for example, be temporary or permanent, moving for specific jobs migrants or other reasons. Their ethnicity and language capabilities will affect their work opportunities.

**Results:**

The occupational health literature seldom adequately identifies the heterogeneity or characteristics of the migrant group being studied. Migrants tend to work in more physically and mentally demanding environments with higher exposures than native workers. Migrants tend to have an increased risk of physical and mental ill health, but less access to health care services. This has been demonstrated recently by high rates of COVID-19 and less access to health care. There have been a number of cross-sectional studies of migrant health but few long-term cohort studies were identified. Other study designs, such as registry-based studies, surveys and qualitative studies may complement cross-sectional studies. Mixed-methodology studies would be valuable in research on migrants’ occupational health. Language and lack of trust are barriers to migrant research participation.

**Conclusion:**

Targeted research, especially longitudinal, identifying how these economically important but often-vulnerable workers can be best assisted is needed. Researchers should identify the characteristics of the migrant workers that they are studying including visa/migration circumstances (temporary, permanent, undocumented), racial and ethnic characteristics, existing skills and language abilities.

## Introduction

Migration is a global phenomenon playing an essential role in the socio-economic development of many countries. Using 2017 data from the United Nations, the International Labour Organization (ILO) has estimated that globally 164 million people (4.7% of all workers) are migrant workers (International Labour Organization (ILO) 2018). Migrant workers contribute to the economies of both the host country, often meeting needs that are not met by the native population; and the country of origin, by remitting financial support to families. Host countries for international migrants are typically high-income countries in North America, Europe, the Middle East and Australia (Hargreaves et al. 2019; Kennedy et al. 2015).

This paper resulted from OMEGA-NET, an EU-funded Network on the Coordination and Harmonization of European Occupational Cohorts (http://omeganetcohorts.eu), COST (European Cooperation in Science and Technology) Action CA16216 (Bodin et al. 2020; Guseva Canu et al. 2021).

Many migrants work in precarious jobs, in difficult and dangerous conditions, perhaps resulting in adverse physical and/or mental health outcomes, such as injury or more recently high rates of COVID-19 (Ahonen et al. 2007; Hargreaves et al. 2019; Moyce and Schenker 2018). Researchers in OMEGA-NET recognized that the occupational health of migrant workers is under-researched.

This paper has the following aims:

1. To clarify definitions of “migrant” used in the occupational health research.
2. To summarize migrant workers’ industry sectors, occupations, and employment conditions.
3. To identify the occupational health and safety services available to migrant workers.
4. To summarize work-related health problems found among migrant workers.
5. To identify the methodological challenges to research into occupational health of migrant workers.
6. To recommend improvements in migrant occupational health research.

## Methods

OMEGA-NET enabled researchers from across Europe and elsewhere to form interest groups to share knowledge and expertise about occupational health research. The Migrant Worker task group consisted of researchers from several European countries and Australia.

We drew on published literature, particularly on two recent systematic reviews of the occupational health of international migrant workers (Hargreaves et al. 2019; Sterud et al. 2018). We identified the types of studies that had been carried out and the major findings in respect of migrant workers’ work sectors and what is known about migrants’ physical and mental health risks related to occupation. This included a summary of migrant workers’ experience of COVID-19.

We situated the available data in the context of the occupational health services typically available to migrant workers and identified gaps in the research. We then systematically evaluated the strengths and weaknesses of the types of study available to investigate the occupational health of migrant workers and made recommendations to strengthen research in this field. The findings of the current position paper were discussed and refined via several virtual meetings by the authors.

## Heterogeneity and lack of definition for “migrant” in research

There have been a variety of studies of migrant occupational health around the world, but the literature lacks a consensus in terminology. The legal status of migrants varies between countries and between migrant groups within a country. However, papers seldom characterize the migration status (reason for migration and legal/visa status) of the individuals under study. In some studies, the definition of ‘migrant’ derives from data collected for purposes other than the research. Understanding migration status is crucial to the interpretation of migrant occupational health studies, as well as for comparison of the findings between studies.

The work circumstances of the migrant population should be made clear. Migrants may be permanent or temporary (seasonal or perhaps for a few years). Black miners, e.g. from Botswana, may work for many years at the same site in South Africa but were not permanent migrants (Steen et al. 1997). Temporary migrants, often experience employment precarity. Some migrants are employed on short-term visas, perhaps tied to specific jobs, for example unskilled domestic workers (Phillipine Statistics Authority 2018). In addition, documented and undocumented (“official vs unofficial”) migrants have very different experiences and vulnerabilities at work (Ahonen et al. 2009).

Within a host country, the migrants’ country of origin and language skills significantly affect the employment experience (Daly et al. 2019; Sole et al. 2013). Skilled and educated workers, such as trained medical practitioners, may migrate and take up suitably skilled jobs (Kizito et al. 2015; Marusic and Markovic-Denic 2018). The skill status of the migrants under study should therefore also be made clear.

Migrants’ ethnic origin, skin colour and religion affect their employment opportunities and work experiences. Some groups encounter more discrimination than others (Balidemaj 2017; European Union Agency for Fundamental Rights 2017). Race and ethnicity are often used interchangeably in the literature, and without being sufficiently defined (Zagefka 2009). Papers from the USA often distinguish risks separately for Black and Caucasian workers and may identify Hispanic or Latino ethnicity (Bahrami et al. 2017; McCurdy et al. 2014). Papers from Europe seldom report on ethnicity or race although this may affect their work experience.

Some papers use a definition of migrant based on language. However, a person’s first language may or may not indicate that the person is a migrant. A definition based on language spoken at home, or the need for an interpreter may result in the inclusion of second-generation migrants rather than first-generation. (Daly et al. 2019).

The term “migrant worker” has also been used to refer to natives moving within a large ethnically and culturally diverse country, perhaps from the countryside to the town (Al-Ayyadhi and Akhtar 2018; Fan and Qian 2017; Zeng et al. 2014). Such internal migrants may or may not experience language and cultural differences; however, if they do, they would be in a comparable situation to many international migrants (Bergbom and Vartia 2021). South African mines relied on a “migrant labour system” which provided employment for men from neighbouring countries as well as from within South Africa (Steen et al. 1997). In a paper on South African miners, it was not always clear whether study participants came from within the country or were international migrants, the term “migrant” was not used (Naidoo et al. 2005). Workers commuting between countries who return home on days off, e.g. Swedes in Norway, have more in common with domestic fly-in-fly-out workers such as Australians traveling to mines in remote areas of Australia than they do with other international migrant workers.

Other terms used in the literature are seasonal, guest or foreign-born worker (Ahonen et al. 2007; Frank et al. 2004; Rebecca Smith 2012). The terms “refugee” or “asylum seeker” identify the reason for the migration. These people are particular subsets of migrants, who have not moved specifically for employment opportunities, but to escape, for example, war or persecution.

Occupational health research should identify the migration status, skills including language skills, and other circumstances of the migrant workers under study, so that readers have a better understanding of the findings of the research, how they should be interpreted and where they can be generalized.

## Characteristics of international migrants’ work

International migrants are commonly employed in construction, agriculture, hospitality, cleaning, transportation, healthcare and personal care sectors (Philippine Statistics Authority 2018; Maji et al. 2020; Ruhs and Anderson 2010). Worldwide studies of migration have shown that the male migrant workers are employed in agriculture, construction, the food sector, transport and material moving occupations, while the majority of migrant women workers are employed in the service sectors, such as domestic work, cleaning, catering, hospitality, and the healthcare and manufacturing sectors (Foley and Piper 2020; González and Irastorza 2007; U.S. Bureau of Labor Statistics 2019).

Migrant workers often display commitment, availability and flexibility, and may accept lower wages and so may be a preferred source of employees. This is notably the case where there are rapid fluctuations in demand and high turnover of unskilled employees for example in seasonal farm work (Chartered Institute of Personnel and Development (CIPD) 2013; Janta et al. 2011).

Migrant workers commonly work in what are known as the 3-D jobs “dirty, dangerous and demanding or demeaning” often characterized by lower pay, longer working hours, more exposure, e.g. to chemicals and no work training (Moyce and Schenker 2018). Many of the industries where migrant workers are employed, have hazards, such as extreme temperatures, noise, vibrations, heavy loads, or fast work speeds (Ronda Pérez et al. 2012). Within an industry or job, migrants may be more highly exposed than native workers (Reid et al. 2018).

Immigration status may affect employment quality, and migrants may be constrained by host country requirements e.g. dependent on a temporary work permit (McDowell 2008). Migrant workers, especially those who have recently arrived, are more likely than their native counterparts to find themselves in short-term, agency and precarious employment. Precarious employment is an important social determinant of health and typically includes employment insecurity, inadequate income and limited rights and protection (Kreshpaj et al. 2020; Siegmann and Schiphorst 2016). They may have limited access to legal expertise, collective bargaining agreements and union representation. Labour market deregulation has particularly affected sectors commonly employing migrant workers, such as construction, agriculture and services (Pajnik 2016). Migrant workers typically have poorer social networks limiting opportunities to find work (Salvatore et al. 2013). Migrant workers are more likely to work in unregulated sections of the labour market and are consequently exposed to social segregation and are vulnerable to exploitation and abuse (Alberti et al. 2013; Bretones et al. 2020; Thornley et al. 2010).

Migrant workers’ employment security, immigration status and labour market policies have a bearing on occupational health and should be identified as well as the industry sector and specific job being researched.

## Migrant access to health and safety services

Migrant workers contribute to the economy of both their origin and host countries, sometimes at a very high personal cost (Moyce and Schenker 2018). It is in the interest of both the receiving country and the country of origin to keep this population healthy, to provide healthy working and living conditions and to allow access to health care.

The healthy migrant effect has been reported in relation to permanent migrants (Kennedy et al. 2015). Migrants, the majority of whom are from developing countries, tended to be healthier than the average of people in the country they migrate to. They were also healthier than those remaining at home perhaps as a result of health screening or selection for skills and education by host country (Kennedy et al. 2015). In some cases, such as Australia, pre-migration health checks are required. Over time, permanent migrants’ health has been shown to deteriorate (Claussen et al. 2009). Some of this change is likely lifestyle-related, for example, metabolic syndrome, alcoholism and diabetes affect the health of recent migrants and are more common among male migrant workers than the native male population (Mucci et al. 2019, 2020). Much is likely to be a result of poor working conditions and less access to health care. The extent to which these changes affect the health status of temporary migrants is unclear.

Interventions to ensure the good health of migrants encompass both occupational health and safety, and primary health care. This includes access to safe and healthy working conditions, targeted medical surveillance, vaccination programs and referral for treatment. Information on hazards, good working practices and control measures, such as ventilation and personal protective equipment, should be provided by the employers in migrants’ language (Hargreaves et al. 2019).

The current literature has little information on what occupational health services are provided to migrants, the quality of the services or the ease of access. The range and quality of services will vary from country to country (or jurisdiction to jurisdiction). Further research is needed to support evidence-informed policy-making to identify which types of health insurance/health provision are best for migrant workers.

A study of the policies/interventions to improve migrant health showed that only 11 of the 25 included EU countries had established specific national actions to improve migrant health beyond statutory legal entitlements (Peiro and Benedict 2009). The report showed that the health services targeting migrant workers usually cover primary health care and occupational health and safety. Preventive services and long-term care did not receive sufficient attention. In particular, the neglected areas were: infectious diseases (including vaccination/immunization), mental health, dental health, sexual and reproductive health, and family health (paediatric services). Migrant workers may receive these services from government authorities (health, labour, immigration or law enforcement) and/or from private or non-governmental organisations (Simon et al. 2015).

Many migrant workers, particularly undocumented workers and those in the black economy, are likely to experience health care inequalities. Entitlement to health care and to effective preventive measures does not necessarily ensure equality of access because there may be other barriers, such as language, physical accessibility, lack of information on how to navigate the health care system, financial cost, etc.

The heterogeneous composition of the migrant worker community, in terms of legal status, qualifications and skills, cultural background, language abilities, labour market integration and work experience leads to a variety of health care needs. A much richer picture of these needs in relation to the sectoral distribution of migrant employment is needed (Ambrosini and Barone 2007). For example, unskilled personnel working in tourism (hotels and restaurants) and household services may need health care to prevent infectious diseases and to control exposure to cleaning materials and disinfectants. For employees in the construction sector, access to emergency health care is necessary. Maternity health services should be considered for all female migrant employees, and child health services may be needed by all migrant workers. Health care services for migrant sex workers are usually restricted to the prevention of sexually transmitted diseases. However, these migrants may also need emergency, reproductive, nutrition and dental care (Global Network of Sex Work Projects NSWP 2018).

## Occupational health outcomes for migrant workers

In the last decade, there has been a significant increase in the number of studies of work-related health problems suffered by migrant workers. Studies in different European countries, and outside Europe showed that migrant workers have high risks of workplace injury, occupational disease, work-related disease and ill health (Abubakar et al. 2018; González and Irastorza 2007; Hargreaves et al. 2019). Although, providing a comparison of work-related health problems between migrant and native workers is often difficult, it has been stated that migrant workers have more absenteeism and sickness leave than native workers. This may be related to higher stress and poorer working conditions (González and Irastorza 2007). They may also exhibit more presenteeism, not taking time off when they are unwell, especially when they have recently migrated. This may be related to the perceived precarity of their work situation. (Agudelo-Suárez et al. 2010).

Physical and chemical exposures can affect the health of migrant workers and lead to respiratory and occupational skin diseases (Arici et al. 2019; Moyce and Schenker 2018). More work-related disease was found among migrant farmworkers, hairdressers, nail salon workers, domestic and healthcare workers and asbestos miners, suggesting perhaps more exposure than for native workers in the same industry (González and Irastorza 2007; Moyce and Schenker 2018; Reid et al. 2018). Ergonomic risks factors, low back pain and work-related musculoskeletal disorders have also been associated with ethnicity and country of origin (Aung et al. 2019; Hoppe et al. 2014; Sterud et al. 2018). Cancer and long-term chronic diseases, such as pneumoconiosis, are difficult to study in migrant populations (Arici et al. 2019; Naidoo et al. 2005) because long-term follow-up is lacking.

Recent research has highlighted the mental health and social well-being problems of migrant workers and shows that migrant workers are more likely to suffer from psychosocial problems and mental disorders than the native workers (Bretones et al. 2020; Daly et al. 2019; Liu et al. 2020). These mental health problems include depression, anxiety, stress, burnout, daytime sleepiness, insomnia, chronic fatigue, and violence (Capasso et al. 2018; Font et al. 2012; Sole et al. 2013). Factors affecting mental health include personal attributes, such as nationality/ethnicity, culture, language barriers; perceptions, such as perceived health risk at work and perceived job satisfaction; and external factors, such as the job demands/stress, working hours, income, working conditions, stress management strategies, a supportive work environment and social inclusion (Capasso et al. 2018; Clouser et al. 2018; Hargreaves et al. 2019; Sterud et al. 2018). A number of studies have found that separation from the family is an important risk factor for poor mental health. (Mucci et al. 2020) Deficient language skills and non-transferability of education and training can also give rise to occupational stress (Ahonen et al. 2007; Daly et al. 2019). A higher education level may have positive effects on health outcomes for migrants but highly educated migrants employed in jobs for which they are overqualified, have poor self-reported health (Espinoza-Castro et al. 2019).

A study in Australia identified that migrants and native workers had largely the same psychosocial job characteristics; but the severity of the job stressors was higher for migrants (Liu et al. 2020). Moreover, migrant and ethnic minority employees have been shown to be more exposed to workplace bullying and social exclusion than native and ethnic majority employees (Bergbom and Vartia 2021; Rosander and Blomberg 2021). Studies have shown that migrants who were living in shelters or barracks had increased mental health problems (Clouser et al. 2018; Moyce and Schenker 2018).

Less research has been identified on occupational biological risks and related health outcomes in migrant workers. There is, however, literature about workers bringing disease with them when they migrate (Arici et al. 2019). Communicable diseases such as TB in health care workers can spread in the work environment, so it is crucial to assess biological health risks particularly in health care setting.

A study in 14 European countries identified that many migrants had a less healthy lifestyles characteristics than natives, e.g. higher smoking rates (Arsenijevic and Groot 2018). Unhealthy behaviours of migrant workers were more likely to be linked to socio-demographic characteristics and cultural background. However, some migrants had a healthier lifestyle, e.g. workers who are practising Muslims avoid unsafe sexual behaviour and alcohol consumption (González and Irastorza 2007; Shaw et al. 2017). Lifestyle behaviours should be considered in future studies of migrant workers’ occupational health.

### Migrant workers and Coronavirus (COVID-19)

Migrant workers have been studied during the COVID-19 pandemic; they are concentrated in high-risk people-facing industries; hence, there is an inequality in workers’ risk of contracting COVID-19 (Maji et al. 2020). During the first wave of COVID-19, health care professionals and transportation workers were found to be at the highest risk in many countries. In addition, in the second wave, food delivery workers, waiters, bartenders, and taxi drivers were found to be at a higher risk than people in other jobs, in all age groups (Magnusson et al. 2021). These industry sectors have high rates of precarious employment and informal work and migrants are over-represented, so they have experienced more layoffs or reductions in working hours (International Labour Organization (ILO) 2020). On the other hand, some workers in sectors, such as healthcare, transportation and warehousing, and social assistance, faced increased workloads exacerbated by high work turnovers (Papadimitriou and Cseres-Gergelyne Blasko 2020).

Also, migrants are over-represented in these industry sectors. Further, migrant workers who live in dormitories or work in crowded workplaces are at increased risk of COVID-19 infection (Gorny et al. 2021; Koh 2020).

During the COVID-19 lockdowns, closed borders, uncertainties about work life, poor working conditions and job insecurity were reported by migrant workers (Greenaway et al. 2020; Roy et al. 2020; Suresh et al. 2020). They faced poorer access to health care; job and wage loss, accommodation insecurity, difficulties in finding basic food and hygiene needs and anxiety about family members living in different countries (Adhikari et al. 2020; Kluge et al. 2020). Migrant health care professionals had a high level of mental distress and poor general health associated with limited access to personal protective equipment (Attal et al. 2020).

Migrants were more likely to be infected with COVID-19 (Koh 2020) and in the UK, the mortality rate for those infected was also higher, especially at the early stage of the outbreak (Aldridge et al. 2020). In addition, migrant workers may be less likely to adhere to protective measures, perhaps increasing the risk of COVID-19 infection (Skogberg et al. 2021). In the current COVID-19 health emergency, migrants need equal access to health care including to vaccination/immunization programs. If they are in people-facing occupations, it is arguable that they should be a high priority in these programs.

## Methodological challenges

Studying the occupational health of migrants poses a variety of methodological challenges. The majority of studies conducted on migrants’ occupational health have been cross-sectional survey studies (Hargreaves et al. 2019; Sterud et al. 2018). Cross-sectional studies can be vulnerable to the Healthy Worker Effect (Fox and Collier 1976). Long-term follow-up is needed to identify excess risk for long-latency occupational diseases, such as cancer or silicosis. Some longitudinal studies were identified including among South African mine workers. Even here authors identify a likely bias “Black miners, upon retiring, return to their distant homes, and are unlikely to have autopsies performed on them.” (Naidoo et al. 2005). This will lead to underestimation of risks. Longitudinal (prospective) studies on migrants present significant logistical problems of follow up especially when studying undocumented, seasonal or temporary migrant workers.

Registry-based studies, surveys and qualitative studies complement each other, each has their own weaknesses and strengths. Table 1 systematically evaluates the strengths and weaknesses of these three study types of study in relation to migrant workers. Because of the different strengths and weaknesses of different research methodologies, mixed-methodology studies would be valuable in research on migrants’ occupational health. Some intervention studies identifying effective strategies to improve migrant health have also been carried out (Sterud et al. 2018).

**Table 1:** Strengths and weaknesses of different types of studies in respect of migrant workers: Registry-based, survey and qualitative studies

	Registry-based studies		Survey studies		Qualitative studies	
	Strengths	Weaknesses	Strengths	Weaknesses	Strengths	Weaknesses
Research design	Longitudinal	Few potential confounders	Prospective studies: causal inferences. Control of some relevant confounders	Cross-sectional: makes causal inferences difficult	Deeper understanding of phenomena Possibility of intervention Possible to study small groups. Include marginal communities Good for minority studies Sensitive to religious and cultural identities	Analysis and interpretation can be time-consuming Researcher must be present during data collection which can bias responses Harder to maintain or demonstrate scientific rigor
Data collection	Register linkage Data are already collected	Cannot influence data collection (type of data, categories etc.)	Good influence on data collection if data collected for the study	May be costly and time-consuming to collect data, particularly of large samples Quality dependent on valid and reliable measures Surveys should be translated and checked for cultural validity Cannot influence data collection if already collected data is used	Recording of interview (if possible) can facilitate a more objective analysis later Data can be collected until the needed saturation point is reached Personal interaction with respondents Builds trust, mutual respect. "Verstehen" Errors with interpretation can be corrected in the course of data collection Sensitive to body language Observation of face mimics, clothes, manners Symbolic interaction In some methods (content analysis) data collection is independent from time, schedules, availability of respondents	Interviews: Dependent on interviewees' ability to verbalise and reflect—may make collection a long process and hard work Success of data collection dependent on Interviewer’s ability to build rapport and trust, and that both can communicate in the same language Ethnographic and observation studies may require much work and time
Data characteristics	“Objective”, not self-reported	Not collected for research purposes May be “crude” or proxy for the data ideally wanted Much work preparing data for analysis	Can be tailored for purpose of study	Subjective i.e. self-reported—> common method with high variance Respondents' lack of trust and/or misunderstandings of survey questions may distort data	Possibility to get data otherwise hard to get (e.g. ethnographic and observation studies) Applicable to groups (rather than only individuals) Revealing in-depth characteristics. Studying phenomena rather than simply facts Reflective, Researcher gains first-hand experience Great sensitivity to language	Data pertains to the sample in question Possible problems of generalizations Subjective Some qualitative methods are idiosyncratic Limited generalizations in time, space and number of people
Sample	Complete for registered migrants Not dependent on language abilities or cultural factors	Undocumented migrants not included May return to home country for medical treatment, or to die if seriously ill (Salmon bias) Loss to follow-up of migrants with poor health	Possible to have representative samples	Contingent on response rates Possibility of systematic self-selection – so non-representative Dependent on respondents' language abilities, literacy, trust and cultural factors Undocumented migrants not included Moonlight work not included Prospective studies: attrition—> loss of those who have become seriously ill, died, fired or returned to home country	Include specific samples: snowball samples, saturation samples, "theoretical" samples (Grounded theory)	Non-representative Small in size Access only to available respondents May have low statistical power
Immigrant background	Available data may include: country of birth, country emigrated from, year of immigration, reason for migration etc	Data may differ between countries	May include: country of birth, country emigrated from, year of immigration, reason for immigration, intentions to relocate or stay in the country, etc	When using already collected data, e.g. population studies, not particularly focusing on migrants: there may be very limited information on immigrant background data	Interviews may include: country of birth, country emigrated from, year of immigration, reason for immigration, intentions to relocate or stay in the country, etc	Ethnographic and observation: data of immigrant background may be very limited or not available
Occupation/ industry	Occupation and industry usually available	Details of specific tasks may be lacking	Available if asked or if survey targeted to special groups. Can collect task-specific information	May lack data not collected for the study purpose	Interviews: may ask about industry/occupation or is known from study selection Individuals describe the job assisting coding to occupation and industry Allows multiple jobs	
Occupational exposures	Job-Exposure Matrices (JEMs) may be linked with occupational titles/codes	No data on occupational exposures	Subjective estimations of asked work conditions Compare to native worker exposures	Lack of data on exposures not easy to estimate	Interviews: Subjective estimations of asked work conditions Ethnographic studies: observation of some work conditions (e.g. observation of whether migrants and non-migrants with same jobs actually do the same tasks)	Lack of reliable measures
Health outcomes	May include: Patient/hospital discharge diagnoses, incl. injuries Cause of death diagnoses Sickness absence/disability diagnoses	Not available Subjective symptoms Wellbeing Job satisfaction	May include subjective: symptoms, well-being, physical and mental health, job satisfaction, work ability, estimates of future work ability, work injuries, estimations of sickness absence, retirement intentions	Not available: death and causes thereof, disability diagnoses. Memory loss—> estimates regarding past time not reliable	Interviews: may include subjective estimations of well-being, physical and mental health, job satisfaction, workability etc	Not available: death and causes thereof Lack of reliable measures
Work-relatedness of health outcomes		Not available	Available to a certain degree		Interviews: Subjective estimations	Interviews: Memory loss and memory change with regard to past times Ethnographic studies: Not available
Work participation	May include: Employment, unemployment, Sickness absence, Presenteeism Disability pension, Retirement (early/old age)		Employment, memories of unemployment Population studies: unemployment	Not available: sickness absence (except for memories of past of those who respond to a survey), disability pension, retirement	Interviews: may include employment, unemployment and their change in time, retirement	Interviews: Memory loss and memory change with regard to past times

Prospective surveys of migrants may be hard to successfully conduct because of low response rates and attrition particularly of temporary workers. Registry-based studies are helpful in this context. The strengths of registry-based studies are that they can be conducted using a longitudinal design, using objectively collected data, which enables causal inferences (Thygesen and Ersbøll 2014). Moreover, registry-based studies are less likely than surveys or qualitative studies to suffer problems of language and cultural barriers. Some Scandinavian countries have national registers which include data on migrants. The shortcomings of registry-based studies include that researchers cannot influence the data that are collected and there is limited ability to control for confounders and they are unlikely to include undocumented migrants. The strength of survey and qualitative studies, are that data collection can be tailored to the research questions of the particular study.

A common and serious problem in surveys of migrants is a low response rate. This can lead to systematic under-representation of migrants or of some migrant groups, resulting in bias and lack of generalizability of findings (Moradi et al. 2010). One obvious cause of low response rates is the language barrier. Poor proficiency in the survey language may lead to misunderstanding of questions and response scales. Thus, survey studies should ideally be translated and back translated to the required language(s). A study by Moradi et al. (2010) showed that translation of the questionnaire increases migrants’ response rates, not only by removal of the language barrier but importantly, because it gives a sense of inclusion to migrant respondents. Translation of surveys must ensure the equivalence of questions and scales in different languages and that they are culturally valid. Construction and translation of good survey instruments for occupational health research around migrant populations, can be time-consuming and costly (Mladovsky 2007). Guidance has been provided for translation of key EU surveys collecting health information (European Commission 2020). Lack of trust in confidentiality may lead to both low participation and an increase in socially desirable responses (Janus 2010). Thus, building of trust is of paramount importance in surveys and in most qualitative studies. Overlooking these reliability and validity issues may seriously affect the quality of survey studies and their findings.

A particular strength of qualitative studies is the increased ability to study marginal groups including undocumented migrants, migrant sex workers and those in the black economy. In qualitative studies, it is possible to build rapport and trust, which may allow studying sensitive issues. Qualitative studies may give deeper and more nuanced understanding of the phenomena being studied but a weakness may be the difficulty of generalizing to other groups.

Finally, it should be emphasized that migrants are not a homogenous group, and so the occupational health of migrants from all relevant groups should be investigated. This would include permanent, temporary and undocumented migrants and migrants from different countries and ethnicities.

## Concluding remarks and recommendations

Many migrant workers experience precarious work, work in hazardous industries and in the least desirable jobs. They are at increased risk of a variety of mental health and social well-being problems, as well as increased risk of workplace accidents, hazardous exposures, discrimination, workplace bullying and, in some circumstances, violence. Indeed, they may be subject to multiple stigmatizations. Recently, healthcare and domestic workers have been working at increased risk of COVID-19; this is sometimes exacerbated by dwelling in dormitory-style accommodation and perhaps by having multiple jobs.

Migrants are not a uniform entity, they can be permanent or temporary, documented or undocumented, tied to specific jobs or not and may regularly return to the same place for seasonal work. The term “migrant” has used in the literature for both domestic and international migrants.

Researchers should provide a clear definition of the migrant population under study, to enable better understanding and future evidence synthesis. At present, demographic information, such as age, sex, etc., is reported, but for migrants, the visa/migration circumstances (temporary, permanent, undocumented), racial and ethnic characteristics, existing skills and language abilities are seldom reported. A description of the health and safety support available in the workplace(s) would be helpful. These factors can affect migrants’ ability to get work, the type of work obtained and experiences encountered at work. It is often unclear which are the most important factors resulting in migrant physical or mental ill health. Mixed methods and intervention studies for these workers, could provide good evidence of factors that might improve their situation.

Sharing improved and translated survey instruments which take cultural considerations into account could improve the research effort.

The largest challenge in studying this group of workers is the ability to do longitudinal research. It is unclear how much ill health is exported when migrant workers return home. Little is known about the rates of cancer and long-term chronic diseases, such as pneumoconiosis, in returning migrant populations. This is an important gap in the evidence base and should be a research priority.

Further research across the globe is urgently required to understand long-term health and safety outcomes and their causes in this often-vulnerable subgroup of the working population to bring about changes in organizational culture to reduce the risks to their health.
